# Salsalate ameliorates metabolic disturbances by reducing inflammation in spontaneously hypertensive rats expressing human C-reactive protein and by activating brown adipose tissue in nontransgenic controls

**DOI:** 10.1371/journal.pone.0179063

**Published:** 2017-06-06

**Authors:** Jaroslava Trnovská, Jan Šilhavý, Ondřej Kuda, Vladimír Landa, Václav Zídek, Petr Mlejnek, Miroslava Šimáková, Hynek Strnad, Vojtěch Škop, Olena Oliyarnyk, Ludmila Kazdová, Martin Haluzík, Michal Pravenec

**Affiliations:** 1 Centre for Experimental Medicine, Institute for Clinical and Experimental Medicine, Prague, Czech Republic; 2 Institute of Physiology, Czech Academy of Sciences, Prague, Czech Republic; 3 Institute of Molecular Genetics, Czech Academy of Sciences, Prague, Czech Republic; 4 Institute of Medical Biochemistry and Laboratory Diagnostics, First Faculty of Medicine, Charles University in Prague and General University Hospital, Prague, Czech Republic; Max Delbruck Centrum fur Molekulare Medizin Berlin Buch, GERMANY

## Abstract

Chronic low-grade inflammation plays an important role in the pathogenesis of insulin resistance. In the current study, we tested the effects of salsalate, a non-steroidal anti-inflammatory drug, in an animal model of inflammation and metabolic syndrome using spontaneously hypertensive rats (SHR) that transgenically express human C-reactive protein (SHR-CRP rats). We treated 15-month-old male transgenic SHR-CRP rats and nontransgenic SHR with salsalate (200 mg/kg/day) mixed as part of a standard diet for 4 weeks. A corresponding untreated control group of male transgenic SHR-CRP and SHR rats were fed a standard diet without salsalate. In the SHR-CRP transgenic strain, salsalate treatment decreased circulating concentrations of the inflammatory markers TNF-α and MCP-1, reduced oxidative stress in the liver and kidney, increased sensitivity of skeletal muscles to insulin action and improved tolerance to glucose. In SHR controls with no CRP-induced inflammation, salsalate treatment reduced body weight, decreased concentrations of serum free fatty acids and total and HDL cholesterol and increased palmitate oxidation and incorporation in brown adipose tissue. Salsalate regulated inflammation by affecting the expression of genes from MAPK signalling and NOD-like receptor signalling pathways and lipid metabolism by affecting hepatic expression of genes that favour lipid oxidation from PPAR-α signalling pathways. These findings suggest that salsalate has metabolic effects beyond suppressing inflammation.

## Introduction

Metabolic syndrome affects more than 25% of individuals in developed countries and is associated with a 2- to 3-fold increased risk of cardiovascular disease and with a 5- to 9-fold increased risk of developing type 2 diabetes [1,2]. Metabolic syndrome is characterised by the clustering of several risk factors, including obesity, insulin resistance, dyslipidaemia and hypertension. Obesity-associated chronic low-grade inflammation is now recognised as an important cause of obesity-induced insulin resistance [3,4], which is also in accordance with the general assumption that immune and metabolic systems are closely interconnected [5–7]. The inflammatory process, including activation of the innate immune system, may be triggered by metabolic derangements caused by metabolic syndrome.

Salsalate, a non-acetylated form of salicylate that belongs among the non-steroidal anti-inflammatory drugs (NSAID), has a weaker inhibitory effect on the enzyme cyclooxygenase compared to other NSAIDs. However, it effectively reduces inflammatory mediators, including interleukin-6 (IL-6), tumour necrosis factor α (TNF-α and C-reactive protein (CRP), possibly through the inhibition of the NF-κB (nuclear factor kappa-light-chain-enhancer of activated B cells) pathway. This mechanism is thought to be responsible for the insulin-sensitising and glucose-lowering effects of salsalate [8].

Recently, Wang et al. [9] demonstrated the presence of diverse aspects of inflammation in rats with diabetes and the anti-inflammatory effects of salsalate. In our previous studies, we derived a new model of inflammation involving metabolic disturbances and target organ damage in spontaneously hypertensive rats (SHR) that express human C-reactive protein (CRP) in the liver (SHR-CRP transgenic rats) [10]. It has been demonstrated that this model is useful for studying the anti-inflammatory and metabolic effects of drugs, including rosuvastatin [11], fumaric acid esters [12], metformin [13] and fenofibrate [14]. In the current study, we tested the hypothesis that salsalate can protect against CRP-induced inflammation and thus ameliorate metabolic disturbances. In addition, the responsible mechanisms were analysed using gene expression profiles in the liver. It was found that salsalate ameliorates insulin resistance and dyslipidaemia by reducing inflammation induced by the human CRP transgene and by activating brown adipose tissue (BAT).

## Materials and methods

### Animals

As described in detail previously [9], transgenic SHR (hereafter referred to as SHR-CRP) were derived using microinjections of SHR fertilized ova with a construct containing cDNA for human CRP under control of the apoE promoter. The objective was to drive expression of the CRP transgene in the liver where CRP is normally produced. To investigate the effects of salsalate on inflammation caused by human CRP, we randomised 15-month-old transgenic SHR-CRP into groups with or without salsalate treatment. We treated 15-month-old male transgenic SHR-CRP with salsalate mixed as part of a standard diet for 4 weeks. The concentration of salsalate in the diet was adjusted to deliver a daily salsalate dose of approximately 200 mg/kg/day, which was estimated using the formula for dose translation based on body surface area (BSA), and is close to the human dosage of about 3 g/day [15,16]. A corresponding untreated control group of male transgenic SHR-CRP was fed a standard diet without salsalate. In a similar fashion, we studied a group of nontransgenic SHR treated with salsalate and an untreated group of nontransgenic SHR controls. In each group, we studied 6–8 animals. We measured daily food intake in each group by subtracting the amount of food remaining in the cage from the measured amount of food provided each day. The average daily food intake for each group was then calculated by averaging all of the daily intake measurements obtained over the entire course of the study. There were no significant differences in food intake among the 4 groups. Body weights of rats were determined using the CJ-3200CE scale in animal mode (ADE, Germany). The rats were housed in an air-conditioned animal facility and allowed free access to chow and water. At the end of the experiments, animals were sacrificed between 8am and 9am by cervical dislocation in a postprandial state, after which tissues were collected for analysis. These experiments were performed in agreement with the Animal Protection Law of the Czech Republic and were approved by the Ethics Committee of the Institute of Physiology, Czech Academy of Sciences, Prague (Permit Number: 66/2014).

### Oral glucose tolerance test (OGTT)

An OGTT was performed using a glucose load of 300 mg/100 g body weight after overnight fasting. Blood was drawn from the tail without anaesthesia before the glucose load (0 min time point) and at 30, 60 and 120 min thereafter.

### Basal- and insulin-stimulated glucose oxidation and glycogen synthesis in skeletal muscle

In order to measure insulin-stimulated incorporation of glucose into CO_2_ and glycogen, soleus muscles were incubated for 2 hours in 95% O_2_ + 5% CO_2_ in Krebs-Ringer bicarbonate buffer, pH 7.4, containing 0.1 μCi/ml of ^14^C-U glucose, 5 mmol/L of unlabelled glucose and 2.5 mg/ml of bovine serum albumin (Sigma, Fraction V, Czech Republic) with or without 250 μU/ml of insulin. All incubations were performed at 37°C in sealed vials in a shaking water bath. After 2 hours, adipose tissue was removed from the incubation medium for glycogen extraction and measurement of incorporation of ^14^C-U glucose into glycogen.

After 2 hours of incubation, 0.3 ml of 1M hyamine hydroxide was injected into the central compartment of the incubation vial and 0.5 ml of 1M H_2_SO_4_ was added to the main compartment to liberate CO_2_. The sealed vessels were incubated for another 45 min., after which the hyamine hydroxide was quantitatively transferred to the scintillation vial containing 10 ml of toluene-based scintillation fluid for radiolabelled glucose incorporation into CO_2_ counting.

### Glucose utilisation in epididymal adipose tissue

Glucose utilisation in adipose tissue was determined *ex vivo* by measuring the incorporation of radioactive ^14^C-U glucose into adipose tissue lipids. Distal parts of epididymal adipose tissue were rapidly dissected and incubated for 2 hours in Krebs-Ringer bicarbonate buffer with 5 mmol/L glucose, 0.1 μCi ^14^C-U glucose/mL (UVVR, Prague, Czech Republic) and 2% bovine serum albumin, with a gaseous phase of 95% O_2_ and 5% CO_2_ in the presence or absence of insulin in incubation media (250 μU/mL). All incubations were performed at 37°C in sealed vials in a shaking water bath. Estimation of ^14^C-glucose incorporation into neutral lipids was performed. Briefly, adipose tissue was removed from the incubation medium, rinsed in saline and immediately put into chloroform. The pieces of tissue were dissolved using a Teflon pestle homogeniser, after which methanol was added (chloroform:methanol 2:1) and lipids extracted at 4°C overnight. The remaining tissue was removed, KH_2_PO_4_ was added and a clear extract was taken for further analysis. An aliquot was evaporated and reconstituted in scintillation liquid and radioactivity was measured by scintillation counting.

### Lipolysis in isolated epididymal adipose tissue

For measurement of basal- and adrenaline-stimulated lipolysis, distal parts of the epididymal adipose tissue were incubated in Krebs-Ringer phosphate buffer containing 3% bovine serum albumin (Sigma, Fraction V, Czech Republic) at 37^°^C, pH 7.4 with or without adrenaline (0.25 μg/ml). The tissue was incubated for 2 hours and concentrations of NEFA and glycerol in the medium were determined.

### Glucose oxidation and incorporation into BAT lipids

Interscapular BAT was dissected and incubated for 2 hours in Krebs-Ringer bicarbonate buffer with 5 mmol/L glucose, 0.1 μCi ^14^C-U glucose/mL and 2% bovine serum albumin, gaseous phase 95% O_2_ and 5% CO_2_. Glucose oxidation was determined in BAT by measuring the incorporation of ^14^C-U glucose into CO_2_, as described above for skeletal muscle. For measurement of incorporation of radiolabelled glucose into lipids, at the end of incubation BAT was removed from media, rinsed in saline and transferred into chloroform:methanol (2:1). Lipids were then extracted and radioactivity measured.

### Palmitate oxidation and incorporation into BAT lipids

Isolated BAT was incubated in Krebs-Ringer bicarbonate buffer with 0.5 μCi/mL of ^14^C-U palmitic acid (Perkin Elmer, USA) complexed with bovine serum albumin (3 mg/mL, fraction V, Sigma) and 0.3 μmol/mL nonradioactive palmitic acid. Otherwise, incubation and measurement conditions were identical to those described above for glucose incorporation.

### Tissue triglyceride measurements

For determination of triglycerides in the liver and soleus muscle, tissues were powdered under liquid N_2_ and extracted for 16 hours in chloroform:methanol, after which 2% KH_2_PO_4_ was added and the solution centrifuged. The organic phase was removed and evaporated under N_2_. The resulting pellet was dissolved in isopropyl alcohol. Triglyceride content was determined by enzymatic assay (Erba-Lachema, Brno, Czech Republic).

### Biochemical analysis

Rat serum CRP and human serum CRP were measured using ELISA kits (Alpha Diagnostics International, San Antonio, USA). Blood glucose concentrations were measured by glucose oxidase assay (Erba-Lachema, Brno, Czech Republic) using tail vein blood drawn into 5% trichloroacetic acid and promptly centrifuged. NEFA concentrations were determined using an acyl-CoA oxidase-based colorimetric kit (Roche Diagnostics GmbH, Mannheim, Germany) and glycerol was determined using an analytical kit from Sigma. Serum triglyceride concentrations were measured using standard enzymatic methods (Erba-Lachema, Brno, Czech Republic). Serum insulin concentrations were determined using a rat insulin ELISA kit (Mercodia, Uppsala, Sweden). Serum IL-6 and TNF-α were measured using rat ELISA kits (BioSource International, Inc., Camarillo, USA). Serum MCP-1 was determined using a kit from eBioscience (Bender MedSystems Biocenter, Vienna, Austria). Eicosanoids were measured using liquid chromatography coupled to mass spectrometry as described previously [17].

### Parameters of oxidative stress

Oxidative stress was measured according to the activities of anti-oxidative enzymes, concentrations of glutathione, the major physiological mechanism response to oxidative stress and concentrations of lipoperoxidation products. The activity of superoxide dismutase (SOD) was analysed using the reaction of blocking nitrotetrazolium blue reduction and nitroformazan formation. Catalase (CAT) activity measurement was based on the ability of H_2_O_2_ and ammonium molybdate to combine to produce a colour complex detected spectrophotometrically. The activity of seleno-dependent glutathione peroxidase (GSH-Px) was monitored by oxidation of gluthathione using Ellman’s reagent (0.01М solution of 5,5'-dithiobis-2 nitrobenzoic acid). The concentration of reduced glutathione (GSH) was determined by the reaction of SH-groups using Ellman’s reagent. Glutathione reductase (GR) activity was measured by the decrease of absorbance at 340 nm using a millimolar extinction coefficient of 6220 M^-1^cm^-1^ for NADPH (using the Sigma assay kit). Lipoperoxidation products were assessed according to concentrations of thiobarbituric acid-reactive substances (TBARS) determined by assaying the reaction with thiobarbituric acid. Concentrations of conjugated dienes were analysed by extraction in the media (heptan:isopropanol = 2:1) and measured spectrophotometric in heptan’s layer [18].

### Gene expression profiles

Total RNA was extracted from the livers of SHR-CRP transgenic and SHR control rats (n = 4 per group) treated with salsalate or with placebo. The quality and concentration of RNA were determined using a NanoDrop 2000 spectrophotometer (Thermo Scientific). RNA integrity was analysed using an Agilent Bioanalyzer 2100. We only included samples deemed as having an intact RNA profile. The Affymetrix GeneChip^®^ Rat Gene 2.0 ST Array System was used for microarray analysis according to the following standard protocol: 100 ng RNA was amplified with an Ambion WT Expression Kit (Applied Biosystems) and 5.5 μg of single-stranded cDNA was labelled and fragmented with GeneChip WT Terminal Labeling and Hybridization (Affymetrix) and then hybridised on the chip according to the manufacturer’s procedure. The analysis was performed in three replicates. Transcription data were MIAME-compliant and deposited in the ArrayExpress database (E-MTAB-5079).

### Gene expression determined by real-time PCR

Total RNA was extracted from the liver using Trizol reagent (Invitrogen) and cDNA was prepared and analysed by real-time PCR testing using QuantiTect SYBR Green reagents (Qiagen, Inc.) on an Opticon continuous fluorescence detector (MJ Research). Gene expression levels were normalised relative to the expression of the peptidylprolyl isomerase A (*Ppia*) (cyclophilin) gene, which served as the internal control. The results were determined in triplicates. Primers used for the validation of differentially expressed genes selected from significant pathways are given in S1 Table.

### Statistical analysis

Our goal was to determine whether the anti-inflammatory effects of salsalate treatment would be greater in the transgenic strain (expressing human CRP) than in the nontransgenic strain. To this end, we used two-way ANOVA to test for treatment x strain interactions and, if present, to determine whether salsalate would inhibit inflammation to a greater extent in the SHR-CRP transgenic strain than in the nontransgenic strain. For variables showing evidence of treatment x strain interaction we used the Holm-Sidak test, which adjusts for multiple comparisons to determine whether the anti-inflammatory effects of salsalate are significant in the nontransgenic SHR strain and the transgenic SHR-CRP strain expressing human CRP. Results are expressed as means ± S.E.M. Gene expression data were preprocessed using the Partek Genomic Suite (Partek Incorporated). Transcription profiles were background-corrected using the RMA method and probe sets were summarised using median polish, then quantile-normalised and variance-stabilised using base-2 logarithmic transformation. Analysis of variance yielded transcripts differentially expressed between analysed samples (within LIMMA) [19]. Storey’s q values [20] were used to select significant differentially expressed genes (q<0.05). All statistical analysis was performed in R and using Bioconductor [21]. To identify significantly perturbed pathways, we performed SPIA (Signalling Pathway Impact Analysis) [22] on KEGG pathways. Genes with P<0.05 were considered differentially transcribed. Where appropriate, the resulting statistical p-values were corrected for multiple testing using the FDR method [23]. The SPIA method considers overrepresentation of differentially expressed genes, the function of every gene in a given pathway and the magnitude of gene expression changes. In addition, SPIA can indicate whether the signal pathway is activated or repressed.

Statistical analysis of gene expression data was performed using the REST XL program, which tests for significance using a randomisation procedure [24].

## Results

### Salsalate reduced inflammation induced by human CRP

**Fig 1:**
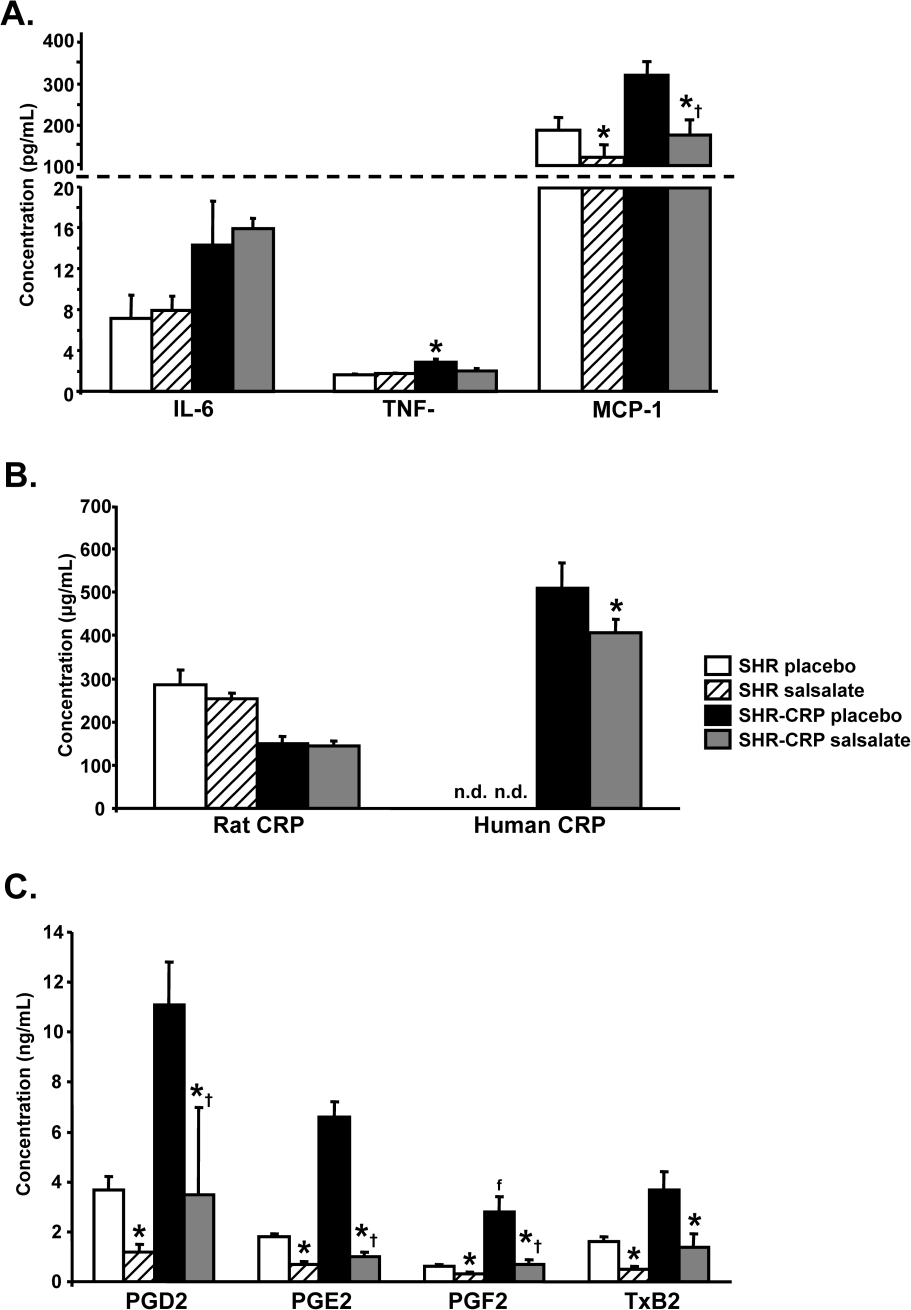
Inflammatory markers in serum from SHR and SHR-CRP transgenic rats treated with placebo or with salsalate. **A.** Concentrations of IL-6, TNF-α and MCP-1 markers. SHR-CRP rats showed significantly increased concentrations of IL-6 when compared to SHR rats, but there were no differences between rats treated with placebo or with salsalate. On the other hand, serum concentrations of TNF-α and MCP-1 in SHR-CRP rats treated with placebo were significantly higher when compared to SHR controls and reduced after treatment with salsalate. No treatment effects were observed in nontransgenic SHR. **B.** SHR-CRP rats treated with placebo or salsalate showed significantly reduced rat endogenous CRP when compared to nontransgenic controls. Transgenic human CRP was significantly reduced in SHR-CRP rats treated with salsalate. **C.** Concentrations of prostaglandins D2, E2, F2α and thromboxane B2 were significantly higher in SHR-CRP rats treated with placebo versus nontransgenic SHR. Salsalate treatment showed a greater decrease in SHR-CRP rats. * denotes significant differences (P<0.05) between placebo versus salsalate-treated groups; † denotes significant strain x treatment interactions, i.e. salsalate treatment protects against the adverse effects of human CRP.

Salsalate treatment was associated with a significant reduction in serum concentrations of TNF-α and MCP-1, while no significant decreases of these inflammatory markers were observed in nontransgenic SHR controls (Fig 1A). As shown in Fig 1B, salsalate reduced serum concentrations of transgenic human CRP but had no effects on serum concentrations of rat endogenous CRP, which were significantly reduced in SHR-CRP transgenic rats when compared to SHR controls. Untreated SHR-CRP transgenic rats had higher serum concentrations of prostaglandins E2, D2, F2α and thromboxane B2 (downstream metabolic products of the cyclooxygenase pathway) when compared to SHR controls. Salsalate treatment significantly reduced concentrations of eicosanoids in both SHR-CRP transgenic and SHR control rats (Fig 1C).

### Salsalate reduced oxidative stress induced by human CRP

As shown in Table 1, activity of the GSH-dependent enzyme GSH-Px (glutathione peroxidase) in the liver and kidney cortex was greater in salsalate-treated SHR-CRP rats but not in SHR controls. Activity of the GSH-regenerating enzyme GR (glutathione reductase) was reduced in the liver of salsalate-treated SHR-CRP rats but the concentration of GSH (reduced glutathione) in tissues remained unchanged. Catalase activity was greater in the liver and aorta in salsalate-treated SHR-CRP and SHR rats compared to untreated controls. Salsalate treatment of SHR-CRP rats was associated with an amelioration of oxidative stress, as the concentrations of lipoperoxidation products measured by TBARS (thiobarbituric acid-reactive substances) and conjugated dienes were lower in the liver and renal cortex. No significant differences were observed in SHR controls (Table 1).

**Table 1:** Parameters of oxidative stress in the liver, aorta and kidney cortex in SHR-CRP transgenic and wild-type controls treated with salsalate or placebo.

Trait	SHR placebo	SHR salsalate	SHR-CRP placebo	SHR-CRP salsalate	P strain	P treatment	P interaction
**Liver**							
SOD (U/mg)	0.129±0.013	0.111±0.007	0.122±0.011	0.116±0.008	NS	NS	NS
GSH-Px (μM NADPH/min/mg)	275±18	287±17	190±18	230±16^*^	NS	NS	0.002
GR (μM NADPH/min/mg)	250±20	306±30	303±22	224±21^*^	NS	NS	0.005
GSH (μM/mg prot.)	43.2±4.5	41.3±2.7	39.2±3.5	39.6±2.1	NS	NS	NS
CAT (mM H_2_O_2_/min/mg)	1246±100	1747±173	1176±99	1520±100^*^	NS	NS	0.039
Conjugated dienes (nM/mg)	35.3±2.7	35.7±3.4	33.6±2.6	31.6±3.9	NS	NS	NS
TBARS (nM/mg)	1.623±0.133	1.434±0.103	2.038±0.158	1.538±0.117^*^	NS	NS	0.004
**Aorta**							
SOD (U/mg)	0.156±0.018	0.186±0.028	0.131±0.014	0.190±0.016	NS	0.03	NS
GSH-Px (μM NADPH/min/mg)	46±4	63±3	30±5	46±4	0.001	0.001	NS
GR (μM NADPH/min/mg)	31±4	28±3	25±2	23±3	NS	NS	NS
GSH (μM/mg prot.)	2.682±0.308	2.242±0.264	2.764±0.562	2.183±0.253	NS	NS	NS
CAT (mM H_2_O_2_/min/mg)	143±15	214±17	180±19	243±23	0.01	0.002	NS
Conjugated dienes (nM/mg)	12.3±1.2	11.7±1.1	16.6±2.1	10.6±0.9^*^	NS	NS	0.02
TBARS (nM/mg)	0.285±0.029	0.264±0.023	0.422±0.029	0.349±0.023	0.001	NS	NS
**Kidney cortex**							
SOD (U/mg)	0.063±0.011	0.051±0.010	0.041±0.005	0.046±0.004	NS	NS	NS
GSH-Px (μM NADPH/min/mg)	150±15	246±15^*^	108±8	146±10^*^	0.001	0.001	0.03
GR (μM NADPH/min/mg)	34±3	48±5	36±2	42±5	NS	0.02	NS
GSH (μM/mg prot.)	15.2±1.5	17.3±1.7	19.2±1.5	19.6±1.1	0.048	NS	NS
CAT (mM H_2_O_2_/min/mg)	509±36	648±53	401±43	374±40	0.001	NS	NS
Conjugated dienes (nM/mg)	14.4±1.2	16.6±2.3	16.4±1.7	11.3±0.6^*^	NS	NS	0.002
TBARS (nM/mg)	0.795±0.028	0.674±0.029	0.951±0.017	0.966±0.049	0.001	NS	NS

Two-way ANOVA results: “P interaction” denotes the significance of salsalate treatment x human CRP interaction (treatment x strain comparison)–salsalate treatment can protect against adverse effects that are dependent on human CRP. “P strain” denotes the significance of SHR-CRP vs. SHR controls (strain effects); “P treatment” denotes the significance of salsalate treatment vs. placebo (treatment effects). For comparisons versus controls, the Holm-Sidak test was used

^*^ denotes P<0.05 significance of comparisons for salsalate vs. placebo treatment of nontransgenic SHR or transgenic SHR-CRP.

NS denotes not significant.

### Salsalate ameliorated insulin resistance induced by human CRP and activated BAT in the absence of inflammation

**Fig 2:**
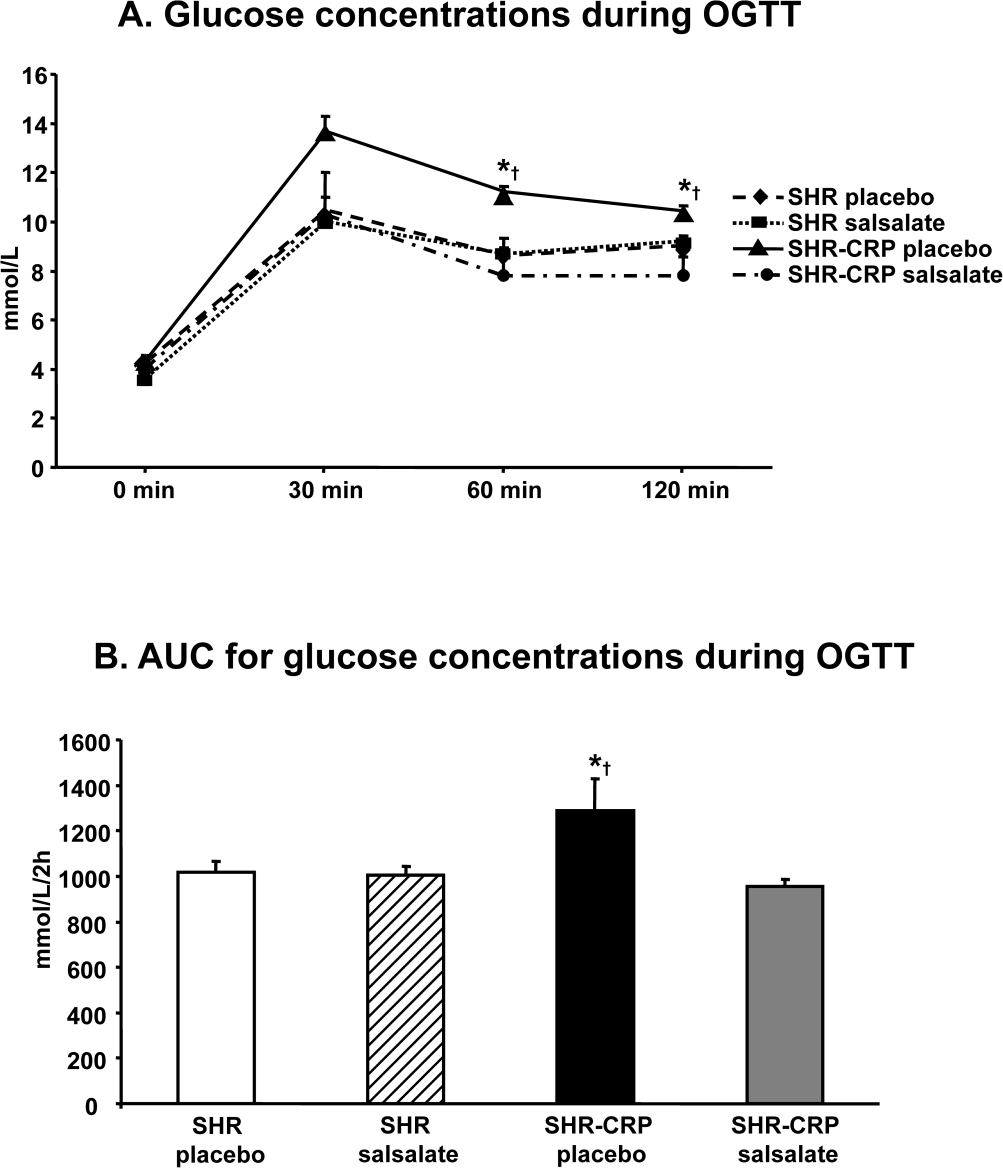
Oral glucose tolerance test in SHR and SHR-CRP transgenic rats treated with placebo or with salsalate. **A.** SHR-CRP transgenic rats treated with salsalate exhibited significantly reduced glucose concentrations (similar to both treated and untreated SHR rats) when compared to SHR-CRP rats treated with placebo. No significant effects of treatment were observed in nontransgenic SHR rats. **B.** Area under the curve (AUC) for glucose concentrations in SHR-CRP rats treated with salsalate was significantly reduced when compared to untreated SHR-CRP rats. * denotes significant differences (P<0.05) between SHR-CRP rats treated with salsalate versus all other groups; † denotes significant strain x treatment interactions, i.e. salsalate treatment protects against the adverse effects of human CRP.

**Fig 3:**
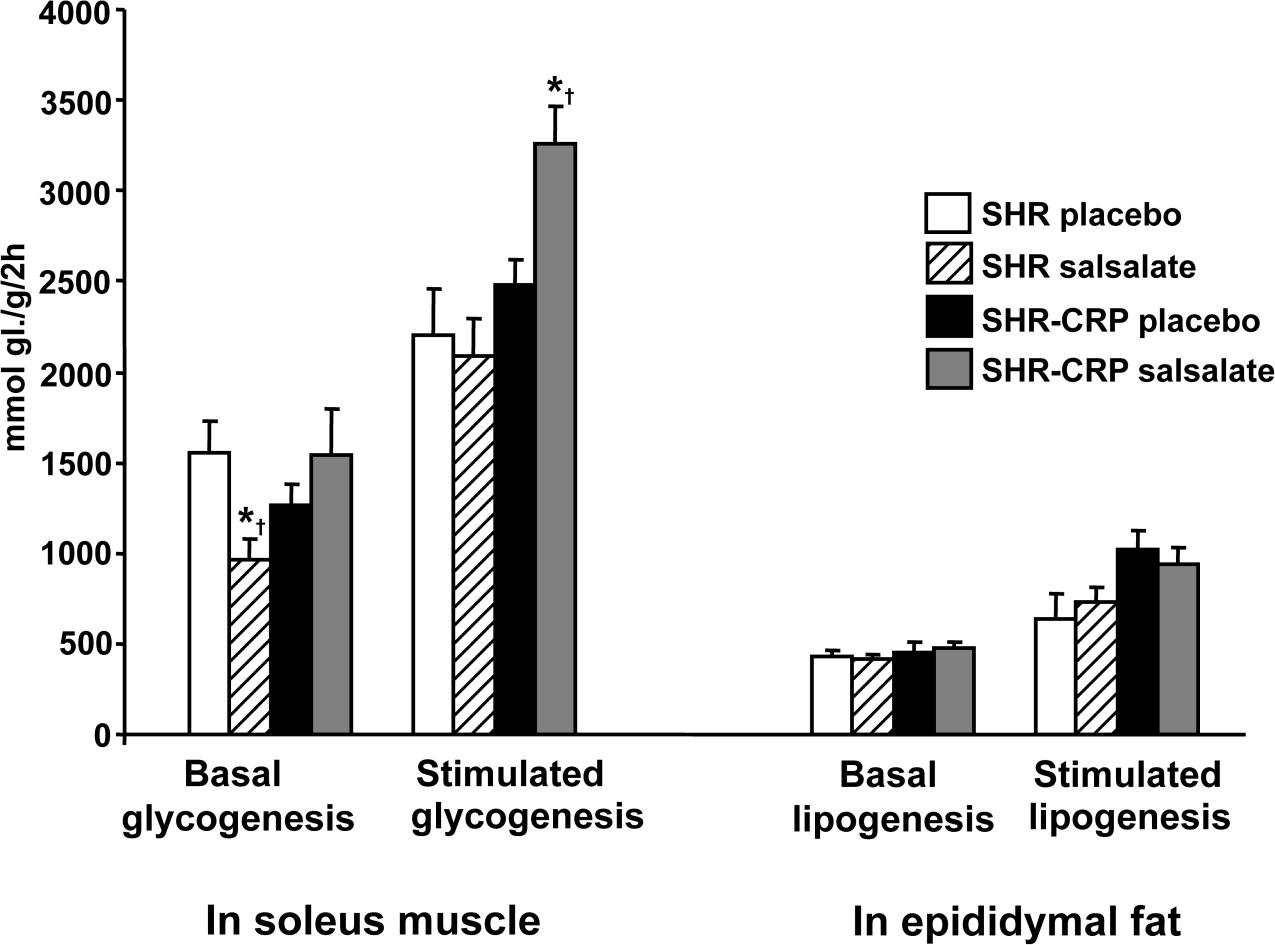
Basal and insulin-stimulated glucose incorporation into skeletal muscle glycogen (glycogenesis) and into lipids in epididymal fat (lipogenesis) in SHR and SHR-CRP rats treated with placebo or with salsalate. SHR-CRP rats treated with salsalate showed significantly increased sensitivity of skeletal muscle to insulin action when compared to nontransgenic SHR. No significant treatment effects were observed for basal- or insulin-stimulated lipogenesis. * denotes significant differences (P<0.05) between placebo versus salsalate-treated groups; † denotes significant strain x treatment interactions, i.e. salsalate treatment protects against the adverse effects of human CRP.

The effects of salsalate on the parameters of insulin resistance were different in the presence of CRP-induced inflammation versus in the absence of inflammation. SHR-CRP transgenic rats treated with salsalate exhibited reduced serum glucose, significant amelioration of glucose intolerance (Fig 2) and higher sensitivity of skeletal muscle to insulin action (Fig 3).

**Fig 4:**
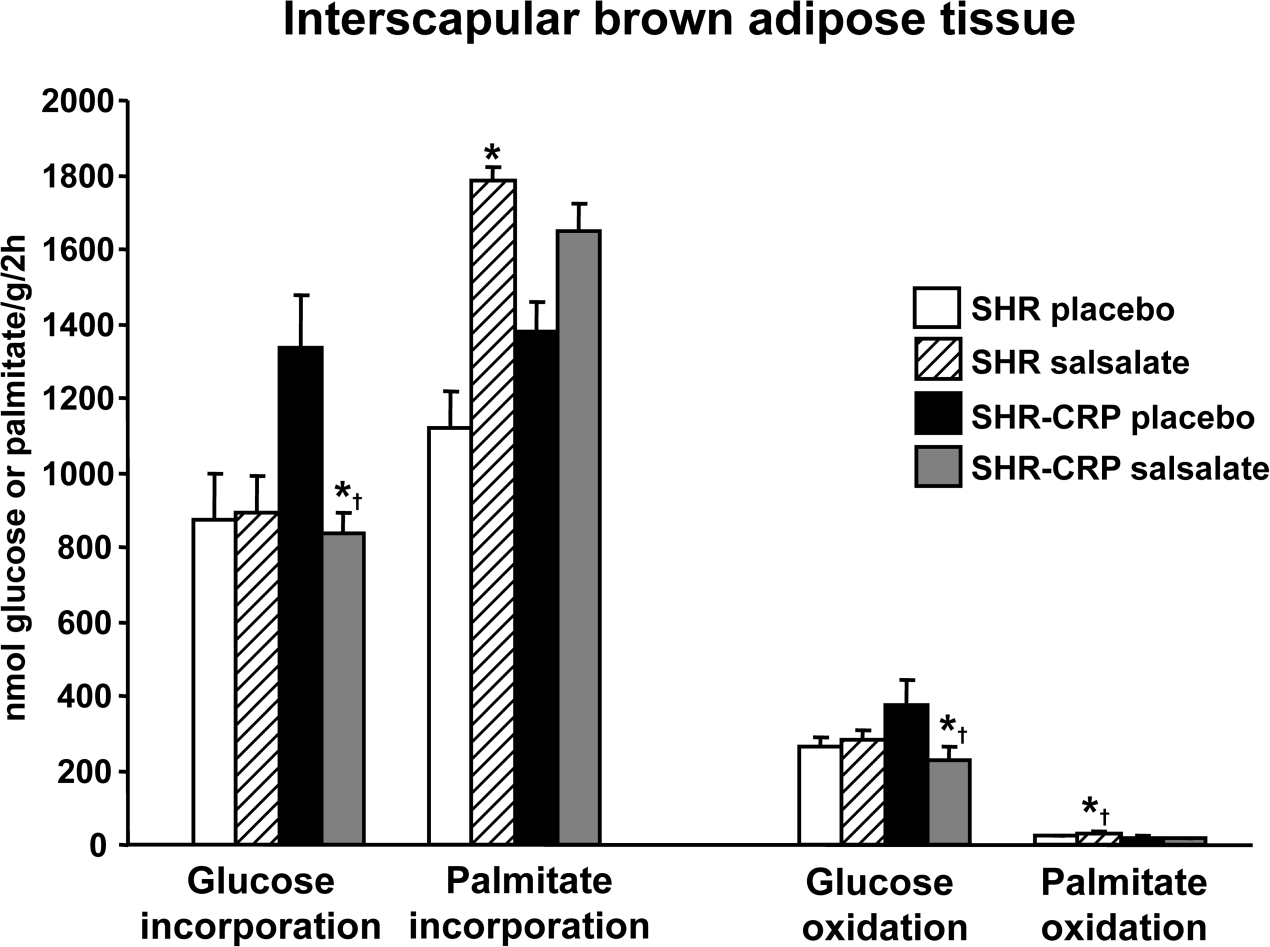
Glucose and palmitate incorporation and oxidation into interscapular brown adipose tissue (BAT) in SHR and SHR-CRP transgenic rats treated with placebo or with salsalate. In nontransgenic SHR, salsalate treatment was associated with increased palmitate oxidation and incorporation into BAT while no significant differences were observed in transgenic SHR-CRP strains after salsalate treatment. On the other hand, SHR-CRP rats treated with placebo had significantly higher glucose oxidation and incorporation into BAT when compared to both SHR-CRP rats treated with salsalate and SHR rats treated with placebo or salsalate. * denotes significant differences (P<0.05) between placebo versus salsalate-treated groups; † denotes significant strain x treatment interactions, i.e. salsalate treatment in the presence of human CRP (in SHR-CRP rats) suppresses glucose oxidation and incorporation into BAT lipids, and increases palmitate oxidation in the absence of human CRP (in SHR controls).

On the other hand, in nontransgenic SHR, salsalate treatment was not associated with an amelioration of insulin resistance. However, it was associated with an amelioration of dyslipidaemia, including reduced serum NEFA, total and HDL cholesterol as well as increased palmitate oxidation and incorporation into BAT lipids (Fig 4). Salsalate treatment increased lipolysis in BAT in both SHR and SHR-CRP rats, but in WAT only in SHR-CRP rats (Table 2).

**Table 2:** Biochemical and metabolic parameters in SHR-CRP transgenic and wild-type controls treated with salsalate or placebo.

Trait	SHR placebo	SHR salsalate	SHR-CRP placebo	SHR-CRP salsalate	P strain	P treatment	P interaction
Body weight (g)	405±6	378±5^*^	366±6	370±7	0.001	NS	0.02
Relative weight of epididymal fat (g/100 g body weight)	0.87±0.03	0.84±0.02	0.62±0.04	0.65±0.02	<0.00001	NS	NS
Relative weight of perirenal fat (g/100 g body weight)	0.80±0.08	0.74±0.05	0.36±0.05	0.35±0.03	<0.00001	NS	NS
Relative liver weight (g/100 g body weight)	3.12±0.06	3.15±0.06	3.93±0.13	3.81±0.120	0.00004	NS	NS
Serum glucose (mmol/L)	6.6±0.1	6.3±0.1	7.3±0.1	6.4±0.2^*^	0.03	0.006	0.043
Serum insulin (nmol/L)	0.263±0.034	0.232±0.030	0.244±0.040	0.228±0.047	NS	NS	NS
Serum triglycerides (mmol/L)	0.59±0.04	0.48±0.04	0.65±0.08	0.56±0.10	<0.00001	NS	NS
Serum NEFA (mmol/L)	0.41±0.04	0.30±0.01^*^	0.31±0.03	0.36±0.03	NS	NS	0.005
Serum cholesterol (mmol/L)	1.57±0.08	1.05±0.03^*^	1.47±0.09	1.48±0.09	<0.05	0.005	0.004
Serum HDL cholesterol (mmol/L)	1.01±0.05	0.66±0.03^*^	0.99±0.03	1.02±0.11	0.03	0.03	0.02
Liver triglycerides (μmol/g)	5.7±0.5	5.4±0.2	12.0±4.1	11.6±4.2	0.04	NS	NS
Muscle gastrocnemius triglycerides (μmol/g)	5.1±0.8	3.2±0.6	4.5±1.0	1.8±0.3	NS	0.002	NS
Lipolysis WAT (NEFA μmol/g)	2.35±0.29	2.37±0.31	2.41±0.15	4.19±0.26^*^	0.003	0.002	0.005
Lipolysis WAT (glycerol μmol/g)	1.14±0.10	1.46±0.09	1.35±0.12	1.81±0.11	0.02	0.001	NS
Fatty acid reesterification	2.05±0.14	1.76±0.27	1.70±0.17	2.36±0.17^*^	NS	NS	0.03
Lipolysis in BAT (NEFA μmol/g)	6.77±1.08	8.25±0.99	5.39±0.43	8.36±0.11	NS	0.04	NS

Two-way ANOVA results: “P interaction” denotes the significance of salsalate treatment x human CRP interaction (treatment x strain comparison)–salsalate treatment can protect against adverse effects that are dependent on human CRP. “P strain” denotes the significance of SHR-CRP vs. SHR controls (strain effects); “P treatment” denotes the significance of salsalate treatment vs. placebo (treatment effects). For comparisons versus controls, the Holm-Sidak test was used

^*^ denotes P<0.05 significance of comparisons for salsalate vs. placebo treatment of nontransgenic SHR or transgenic SHR-CRP.

NS denotes not significant.

### Gene expression profiling

**Fig 5:**
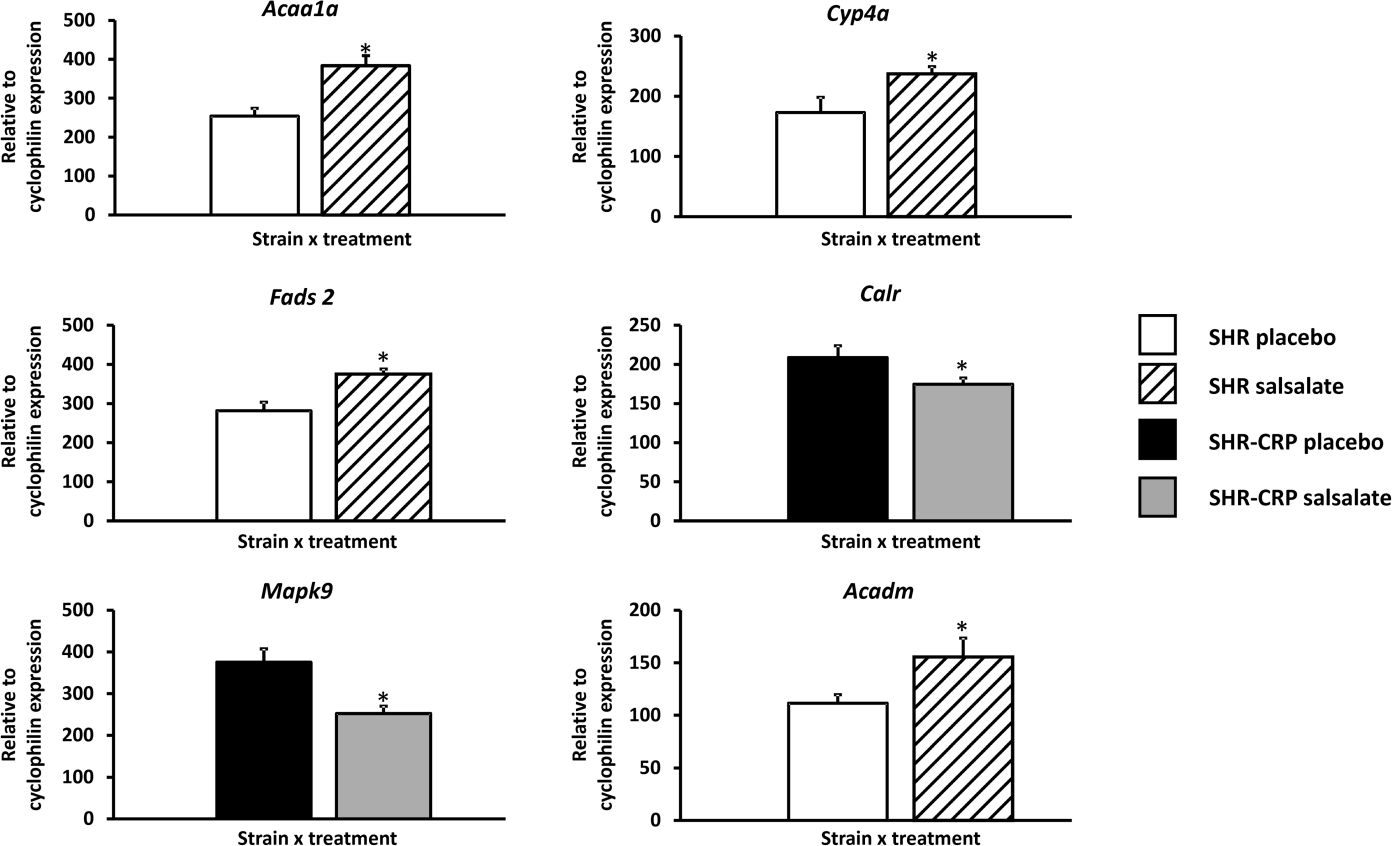
Validation of directional expression of genes identified by SPIA using quantitative real-time PCR for six transcripts in livers isolated from SHR-CRP rats versus SHR controls treated either with placebo or salsalate. The expression of selected genes was normalised relative to the expression of the peptidylprolyl isomerase A (*Ppia*) (cyclophilin) gene, which served as an internal control. * denotes p<0.05 and p<0.001.

To search for the molecular mechanisms responsible for the effects of salsalate on inflammation, oxidative stress and metabolic disturbances associated with transgenic expression of human CRP, we performed genome-wide expression profiling in the liver isolated from SHR and SHR-CRP strains treated with salsalate or a placebo. Altogether, we detected more than 90 genes showing genome-wide significant differential expression (q<0.05) (S2 Table), while 2619 genes were differentially expressed at a nominal p<0.05 value. The effects of salsalate on hepatic gene expression levels in the SHR-CRP transgenic strain were similar to those in the nontransgenic strain. Although we did not detect any significant treatment x strain interactions, we observed significant salsalate treatment effects. Table 3 displays the genes from KEGG pathways identified by SPIA, including PPAR signalling, circadian rhythms and protein processing in endoplasmic reticulum pathways. These pathways include deregulated genes involved in lipid metabolism, including the fatty acid metabolism pathway (*Acaa1a*, *Acaa1b*, *Acsl4*, *Acadl*, *Acadm*, *Acox1*, *Cpt2*, and *Ehhadh*), the biosynthesis of unsaturated fatty acids pathway (*Acaa1a*, *Acaa1b*, *Acox1*, *Fads2*, and *Scd1*), the valine, leucine and isoleucine degradation pathway (*Hmgcs2*, *Acaa1a*, *Acaa1b*, *Acadm*, and *Ehhadh*), the adipocytokine signalling pathway (*Acsl4*, *Ppara*, and *Pck1*) as well as genes involved in inflammation, including the antigen-processing and presentation pathway (*Calr*, *Hspa2*, *Hspa8*, *Hsp90ab1*, and *Pdia3*), the MAPK signalling pathway (*Ddit3*, *Traf2*, *Hspa2*, *Hspa8*, and *Mapk9*) and the NOD-like receptor signalling pathway (*Hsp90ab1*, *Hsp90b1*, and *Mapk9*). Genes involved in inflammatory processes were differentially expressed after salsalate treatment only in SHR-CRP transgenic rats, not in nontransgenic controls. In addition, genes coding for noncatalytic regulatory β1 and γ2 subunits of AMP-activated protein kinase (AMPK) (*Prkab1* and *Prkag2*) and the catalytic α1 subunit (*Prkaa1*) were also found to be deregulated in rats treated with salsalate. The directional expression of randomly selected genes from Table 3 was confirmed using real-time PCR (Fig 5).

**Table 3:** List of genes from KEGG pathways identified by SPIA showing the effects of salsalate versus placebo in SHR controls (A) and in SHR-CRP rats (B).

**A. Effects of salsalate vs. placebo in SHR controls**	**FWER**	**Deregulated genes (P<0.05)**
PPAR signalling	2.15e-06	↑*Ehhadh*, ↑*Acaa1a*, ↑*Acaa1b*, ↑*Fads2*, ↓*Cyp4a8*, ↑*Cyp4a1*, ↑*Acsl1*, ↓*Apoa5*, ↑*Acadl*, ↑*Me1*, ↑*Acsl4*, ↑*Slc27a2*, ↑*Fabp1*, ↑*Acadm*, ↑*Acox1*, ↑*Scd*, ↑*Dbi*, ↑*Fabp7*, ↑*Ppara*, ↑*Cpt2*, ↓*Mmp1*, ↑*Cyp27a1*, ↑*Scd1*
Circadian rhythms	0.0014	↓*Arnlt*, ↑*Per3*, ↑*Cry2*, ↑*Per1*, ↑*Skp1*, ↓*Npas2*, ↑*Per2*, ↑*Prkag2*, ↑*Prkaa1*, ↓*Prkab1*
**B. Effects of salsalate vs. placebo in SHR-CRP rats**	**FWER**	**Deregulated genes (P<0.05)**
PPAR signalling	1.49e-05	↑*Acaa1b*, ↑*Ehhadh*, ↑*Acaa1a*, ↑*Cpt2*, ↑*Acox1*, ↑*Acadl*, ↓*Fabp2*, ↑*Cyp4a1*, ↓*Apoa5*, ↑RGD1565355 (similar to *Cd36*), ↑*Hmgcs2*, ↑*Slc27a2*, ↑*Acadm*, ↓*Ppard*, ↑*Scd1*, ↑*Fabp1*, ↑*Fabp7*, ↓*Cyp4a8*, ↑*Ppara*, ↑*Acsbg1*, ↑*Pck1*, ↑*Acsl4*, ↑*Pltp*, ↑*Fads2*
Circadian rhythms	5.49e-05	↑*Cry1*, ↓*Npas2*, ↑*Per2*, ↓*Prkab1*, ↑*Per3*, ↑*Per1*, ↑*Cry2*
Protein processing in the endoplasmic reticulum	0.0036	↑*Hsph1*, ↑*Hspa8*, ↑*Hsp90ab1*, ↑*Ddit3*, ↑*Dnaja1*, ↓*Uggt1*, ↓*Calr*, ↑*Sec23b*, ↓*Pdia6*, ↓*Pdia3*, ↑*Herpud1*, ↓*Xbp1*, ↓*P4hb*, ↓*Rpn1*, ↑*Ufd1l*, ↓*Dnajc3*, ↓*Hsp90b1*, ↓*Stt3b*, ↑*Plaa*, ↓*Dnajb11*, ↑*Hspa4l*, ↑*Dnajb2*, ↑*Eif2s1*, ↑*Vimp*, ↓*Sel1l*, ↓*Sec61b*, ↓*Derl3*, ↓*Os9*, ↓*Ddost*, ↓*Pdia4*, ↓*Fbxo6*, ↓*Tram1*, ↑*Nploc4*, ↑*Hspa2*, ↓*Mapk9*, ↑*Traf2*

↑ and ↓ denote up- and downregulated, respectively, in salsalate- versus placebo-treated rats. FWER–Family-Wise Error Rate

## Discussion

In the current study, we tested the effects of salsalate on inflammation, oxidative stress and glucose and lipid metabolism in a model of inflammation and metabolic syndrome using SHR-CRP rats expressing human CRP and nontransgenic SHR controls to assess whether salsalate treatment protects against adverse effects that are dependent on human CRP. Our results demonstrate that salsalate significantly reduced inflammation and oxidative stress in SHR-CRP rats and was associated with the amelioration of insulin resistance in skeletal muscle and increased glucose tolerance.

On the other hand, in nontransgenic SHR rats (with significantly lower concentrations of inflammatory biomarkers) salsalate treatment had no effects on inflammation and oxidative stress. However, it was associated with reduced circulating and skeletal muscle lipids and with the activation of BAT, which exhibited increased palmitate oxidation and incorporation. These latter results for nontransgenic SHR rats are congruent with recently published findings in E3L.CETP mice (APOE*3-Leiden.CETP transgenic mice, a well-established model for human-like lipoprotein metabolism), which report that salsalate treatment attenuates and reverses high-fat diet-induced weight gain due to the activation of BAT but exerts no anti-inflammatory effects [25]. To investigated whether salsalate directly activates brown adipocytes and the underlying mechanisms, differentiated T37i adipocytes were treated with salsalate which was associated with increased uncoupled respiration and a dose-dependent increase in *Ucp1* expression [25].

High doses of salicylates reversed hyperglycemia, hyperinsulinemia, and dyslipidemia in insulin resistant Zucker *fa/fa* obese rats by sensitising insulin signalling [26]. To search for responsible mechanisms, 3T3-L1 adipocytes and Fao hepatoma cells were treated with TNF-α which was associated with reduced insulin signalling and with activation of IkB kinase β (IKKβ) (a part of the upstream NF-κB signal transduction cascade) and this was reversed by prior treatment of cells with high doses of aspirin. It has been suggested that the IKKβ pathway is a target for insulin sensitising effects of salsalate [26].

To search for the molecular mechanisms responsible for the anti-inflammatory and metabolic effects of salsalate in SHR-CRP rats, we determined gene expression profiles in the liver. Transcriptomics analysis revealed significantly deregulated genes from the fatty acid metabolism pathway when genes involved in beta-oxidation (*Acaa1a*, *Acaa1b*, *Acsl4*, *Acadl*, *Acadm*, *Acox1*, *Cpt2*, *Ehhadh*, *Ppara*, *Ppard*) were all upregulated in rats treated with salsalate. In addition, we observed differentially regulated *Cyp4a8* and *Cyp4a1* genes coding for cytochromes 4A8 and 4A1 that metabolise EETs (epoxyeicosatrienoic acids) to HEETs (hydroxy-epoxyeicosatrienoic acids) and act as PPAR-α agonists [27] (Table 3). PPAR-α is a major regulator of lipid metabolism in tissues that oxidise fatty acids, such as BAT, the liver and the heart, and is upregulated in rats treated with salsalate. Recently, salsalate has also been reported to directly activate AMP-activated protein kinase (AMPK), which plays an important role in the regulation of inflammation, liver lipid metabolism and mitochondrial uncoupling in BAT [28]. Upon activation, AMPK stimulates catabolic pathways (fatty acid oxidation, glucose transport, etc.). In the current study, we observed increased expression of genes coding for catalytic α1 and regulatory γ2 subunits of AMPK. These results suggest that in SHR nontransgenic rats, in the absence of inflammation associated with the expression of human CRP, salsalate also acts by activating fatty acid catabolism via activation of PPAR-α by ligands derived from the metabolism of arachidonic acid and by regulating AMPK. On the other hand, activation of AMPK by salicylate is controversial since the beneficial effects of salicylate have also been observed in AMPK knockout mice and other mechanisms, including the protonophoric effects of salsalate in tissues relevant to the pathogenesis of metabolic syndrome such as the liver, skeletal muscle and white adipose tissue [29].

Interestingly, the anti-inflammatory effects of salsalate were significant in SHR-CRP rats but not in nontransgenic SHR rats. This is an important and novel observation that may point to the higher efficacy of salsalate in the presence of more pronounced inflammation. These findings may have implications for the potential use of salsalate as an anti-inflammatory treatment in patients with diabetes and metabolic syndrome. It is tempting to speculate that the higher efficacy of salsalate might be expected in subjects with a more pronounced inflammatory response.

Contrary to SHR-CRP, in nontransgenic SHR rats, salsalate treatment was associated with activation of BAT. We can only speculate that inflammation somehow interferes with BAT activation in SHR-CRP rats but not in nontransgenic SHR. For instance, in our recent report [30], we showed that expression of transgenic resistin was associated with activation of the pro-inflammatory response and reduced palmitate and glucose oxidation in BAT in resistin transgenic rats. In addition, Mráček et al. [31] demonstrated that chronic treatment of mouse brown adipocytes in a primary culture with IL-1β, IL-6 and LPS inhibited the maturation of brown adipocytes.

Together, these findings suggest that salsalate has metabolic effects beyond suppressing inflammation. Such pleiotropic effects of salsalate have been observed in both animal and human studies. For instance, Liang et al. [16] demonstrated that salsalate treatment was associated with the amelioration of non-alcoholic fatty liver disease, reduced expression of genes from pro-inflammatory pathways and increased expression of genes from lipid metabolism and energy production pathways. In addition, Smith et al. [29] demonstrated the protonophoric effects of salsalate, which may also explain its beneficial metabolic effects.

Salsalate treatment was also associated with deregulated genes from the circadian rhythms pathway (Table 3). It has been reported that salsalate competes with thyroid hormones by binding to their transfer proteins, displaces T3 and T4 and causes chronic hypothyroidism [32]. Reduced concentrations of thyroid hormones might alter rhythmic expression of circadian clock genes in the brain [33] and further affect circadian rhythmicity in the liver and the metabolism of BAT [34].

The effects of anti-inflammatory therapy in patients with type 2 diabetes have been reviewed recently [35,36]. Specifically, patients with type 2 diabetes in randomised control trials, treated with daily doses of 3–4.5 g salsalate, exhibited amelioration of insulin resistance and reduced glucose, triglyceride and free fatty acid concentrations [37–39]. However, the observed effects on HbA1c in these trials were rather modest (an approximate 0.3% reduction).

In summary, the results of the current study demonstrated the pleiotropic effects of salsalate: (1) anti-inflammatory effects due to reduction of downstream metabolic products of the cyclooxygenase pathway (pro-inflammatory prostaglandins and thromboxanes) and (2) hypolipidaemic effects due to increased fatty acid oxidation induced in BAT possibly via activation of PPAR-α by ligands derived from the metabolism of arachidonic acid and through regulation of AMPK. These effects of salsalate were associated with the amelioration of insulin resistance, glucose intolerance and with protection against oxidative stress in target organs. The reasons for the more pronounced effects of salsalate in experimental studies in comparison with the rather modest effects of anti-inflammatory therapy on cardiovascular disease reduction and glucose control in humans need to be further elucidated. Our results suggest that salsalate treatment may be especially effective in patients with higher concentrations of CRP.

## Supporting information

S1 Table Primers for validation of directional expression of genes identified by gene expression profiling. (DOC)

S2 Table Genes showing genome-wide significant differential expression (q<0.05). (DOC)
