# Supplementary material for: Salsalate ameliorates metabolic disturbances by reducing inflammation in spontaneously hypertensive rats expressing human C-reactive protein and by activating brown adipose tissue in nontransgenic controls
Source: PLoS One. 2017 Jun 6;12(6):e0179063. doi: 10.1371/journal.pone.0179063 (PMC5460879; doi:10.1371/journal.pone.0179063)
Supplement: S1 Table — (DOC) [file pone.0179063.s001.doc]

**Supplementary Table 1** Primers for validation of directional expression of genes identified by gene expression profiling.

| **Genes** | **Forward primers** | **Reverse primers** |
| --- | --- | --- |
| *Acaa1a* | cacacccagagagccaaga | cctcgctctcttctgttggt |
| *Cyp4a1* | CAAGGTCCCCATCCCCTTAC | CGGAGCTCCACAACGGAAT |
| *Fads2* | TCGACCGCAAGGTCTACAAC | GGAAGGCATCCGTAGCATCT |
| *Calr* | TCATGTTTGGTCCGGACATCT | AGCACGTTCTTGCCCTTGTA |
| *Mapk9* | AGCATGCGATTGAAGAGTGGA | ACTGCTGCATCTGAAGGCTG |
| *Acadm* | ATTCCGGAGAGTTGTGGTGG | TAGCAGTCTGCACCCCTGTA |
| *Ppia* | agcatacaggtcctggcat | tcaccttcccaaagaccac |
