# Supplementary material for: Salsalate ameliorates metabolic disturbances by reducing inflammation in spontaneously hypertensive rats expressing human C-reactive protein and by activating brown adipose tissue in nontransgenic controls
Source: PLoS One. 2017 Jun 6;12(6):e0179063. doi: 10.1371/journal.pone.0179063 (PMC5460879; doi:10.1371/journal.pone.0179063)
Supplement: S2 Table — (DOC) [file pone.0179063.s002.doc]

**Supplementary Table S2** Genes showing genome-wide significant differential expression (q<0.05).

A. In untreated SHR versus SHR-CRP rats

| **Gene symbol** | **Gene name** | **Log FC** | **P value** |
| --- | --- | --- | --- |
| Cyp17a1 | cytochrome P450, family 17, subfamily a, polypeptide 1 | -2.08 | 7.4e-07 |
| Slc3a1 | solute carrier family 3, member 1 | 1.13 | 5.4e-07 |
| Cdh17 | cadherin 17 | 1.63 | 1.6e-06 |
| Echdc1 | enoyl CoA hydratase domain containing 1 | 0.9 | 1.2e-05 |
| Adam8 | ADAM metallopeptidase domain 8 | 2.5 | 9.5e-06 |
| Ccng1 | cyclin G1 | 0.9 | 1.1e-05 |
| Hsd17b13 | hydroxysteroid (17-beta) dehydrogenase 13 | -1.18 | 3.7e-06 |
| Tspan33 | tetraspanin 33 | 0.58 | 1.3e-05 |
| Gprin3 | GPRIN family member 3 | -2.39 | 4.9e-06 |
| Spa17 | sperm autoantigenic protein 17 | 0.83 | 9.8e-06 |
| Serpina7 | serpin peptidase inhibitor, clade A (alpha-1 antiproteinase, antitrypsin), member 7 | 2.29 | 7.5e-06 |
| Rap2c | RAP2C, member of RAS oncogene family | 0.73 | 9.5e-06 |
| Tle2 | transducin-like enhancer of split 2 (E(sp1) homolog, Drosophila) | -0.65 | 1.8e-05 |
| Sdf2l1 | stromal cell-derived factor 2-like 1 | 1.48 | 2.5e-05 |
| Pspc1 | paraspeckle component 1 | 0.64 | 2.4e-05 |
| Gdnf | glial cell derived neurotrophic factor | -1.18 | 2.1e-05 |
| Dab1 | Dab, reelin signal transducer, homolog 1 (Drosophila) | -0.9 | 2.6e-05 |
| Ehd3 | EH-domain containing 3 | -0.83 | 2.4e-05 |
| Creb3l3 | cAMP responsive element binding protein 3-like 3 | -0.68 | 2.3e-05 |
| Aspa | aspartoacylase | 0.92 | 3,00E-05 |
| Pnpla3 | patatin-like phospholipase domain containing 3 | 1.17 | 3,00E-05 |
| Lyplal1 | lysophospholipase-like 1 | 0.71 | 4.2e-05 |
| Sod3 | superoxide dismutase 3, extracellular | -0.77 | 4.3e-05 |
| Limk2 | LIM domain kinase 2 | 0.58 | 4.6e-05 |
| Gsta3 | glutathione S-transferase alpha 3 | 1.76 | 4.1e-05 |
| Hhex | hematopoietically expressed homeobox | 1.13 | 6.6e-05 |
| Guf1 | GUF1 GTPase homolog (S. cerevisiae) | 0.65 | 6.6e-05 |
| Shisa3 | shisa family member 3 | -0.63 | 6,00E-05 |
| Klkb1 | kallikrein B, plasma 1 | 0.48 | 5,00E-05 |
| Gstt2 | glutathione S-transferase, theta 2 | 0.56 | 5.1e-05 |
| Itga6 | integrin, alpha 6 | 0.74 | 6.1e-05 |
| Bmp2 | bone morphogenetic protein 2 | -0.78 | 5.1e-05 |
| Slco1a2 | solute carrier organic anion transporter family, member 1A2 | 0.65 | 6.2e-05 |
| Epha2 | Eph receptor A2 | -0.62 | 5.8e-05 |
| Inhbe | inhibin beta E | -1.73 | 5.7e-05 |
| Inhbc | inhibin beta C | -1.04 | 5.8e-05 |
| Pc | pyruvate carboxylase | -0.55 | 7.6e-05 |
| Lyve1 | lymphatic vessel endothelial hyaluronan receptor 1 | -0.68 | 8.4e-05 |
| Kng1l1 | kininogen 1-like 1 | 0.86 | 7.5e-05 |
| Acss2 | acyl-CoA synthetase short-chain family member 2 | 0.87 | 7.9e-05 |
| Hsp90b1 | heat shock protein 90, beta, member 1 | 0.75 | 8.1e-05 |
| Scaper | S-phase cyclin A-associated protein in the ER | 0.66 | 8.1e-05 |
| Pdia3 | protein disulfide isomerase family A, member 3 | 0.57 | 8.7e-05 |
| Fam92a1 | family with sequence similarity 92, member A1 | 0.61 | 9.4e-05 |
| Slc25a47 | solute carrier family 25, member 47 | -1.04 | 9.1e-05 |
| Tmem123 | transmembrane protein 123 | 0.44 | 9,00E-05 |

B. In SHR versus SHR-CRP rats treated with salsalate.

| **Gene symbol** | **Gene name** | **Log FC** | **P value** |
| --- | --- | --- | --- |
| Bag3 | Bcl2-associated athanogene 3 | 0.97 | 2.2e-05 |
| Serpinh1 | serpin peptidase inhibitor, clade H (heat shock protein 47), member 1, (collagen binding protein 1) | 0.56 | 3.5e-05 |
| Adam8 | ADAM metallopeptidase domain 8 | 1.84 | 3.4e-05 |
| Zfand2a | zinc finger, AN1-type domain 2A | 1.85 | 7,00E-06 |
| Hspb8 | heat shock protein B8 | 0.8 | 1.1e-05 |
| Uspl1 | ubiquitin specific peptidase like 1 | 1.22 | 2.6e-05 |
| Hspb1 | heat shock protein 1 | 2.3 | 2.7e-07 |
| Hsd17b13 | hydroxysteroid (17-beta) dehydrogenase 13 | -1.17 | 4.1e-06 |
| Hmox1 | heme oxygenase (decycling) 1 | 1.62 | 2.6e-07 |
| Dnajb1 | DnaJ (Hsp40) homolog, subfamily B, member 1 | 1.31 | 5.2e-07 |
| Ppid | peptidylprolyl isomerase D | 0.65 | 1.9e-05 |
| Dnajb4 | DnaJ (Hsp40) homolog, subfamily B, member 4 | 0.93 | 1.1e-05 |
| H2afj | H2A histone family, member J | 0.6 | 2.3e-05 |
| Slc30a3 | solute carrier family 30 (zinc transporter), member 3 | -0.94 | 1.5e-05 |
| Slc36a4 | solute carrier family 36 (proton/amino acid symporter), member 4 | -0.6 | 3.2e-05 |
| Enpp5 | ectonucleotide pyrophosphatase/phosphodiesterase 5 | 0.67 | 2.9e-05 |
| P4ha1 | prolyl 4-hydroxylase, alpha polypeptide I | 1.22 | 1.4e-05 |

C. In SHR-CRP treated with salsalate versus SHR-CRP treated with placebo

| **Gene symbol** | **Gene name** | **Log FC** | **P value** |
| --- | --- | --- | --- |
| Sertad3 | SERTA domain containing 3 | 1.24 | 6.1e-07 |
| Fus | fused in sarcoma | -0.85 | 7.3e-05 |
| Bag3 | Bcl2-associated athanogene 3 | 1.16 | 2.8e-06 |
| Vnn1 | vanin 1 | 2.26 | 1.7e-05 |
| Serpinh1 | serpin peptidase inhibitor, clade H (heat shock protein 47), member 1, (collagen binding protein 1) | 0.59 | 2.1e-05 |
| Stip1 | stress-induced phosphoprotein 1 | 1.17 | 6.3e-07 |
| Tenm2 | teneurin transmembrane protein 2 | -0.84 | 5.9e-06 |
| Hsph1 | heat shock 105/110 protein 1 | 2.2 | 2.2e-07 |
| Zfand2a | zinc finger, AN1-type domain 2A | 1.83 | 8.1e-06 |
| Hspb8 | heat shock protein B8 | 1.6 | 3.1e-07 |
| Uspl1 | ubiquitin specific peptidase like 1 | 1.13 | 6.3e-05 |
| Hspb1 | heat shock protein 1 | 2.35 | 3.7e-08 |
| Tbx3 | T-box 3 | 1.24 | 3.4e-05 |
| Cacybp | calcyclin binding protein | 0.99 | 1.1e-05 |
| G0s2 | G0/G1switch 2 | 1.16 | 2.6e-05 |
| Agpat9 | 1-acylglycerol-3-phosphate O-acyltransferase 9 | 0.87 | 3.7e-05 |
| Ahsa2 | AHA1, activator of heat shock protein ATPase 2 | 1.15 | 5.3e-06 |
| Hspa8 | heat shock 70kDa protein 8 | 0.65 | 1.5e-06 |
| Ndst1 | N-deacetylase/N-sulfotransferase (heparan glucosaminyl) 1 | -0.97 | 3.5e-05 |
| Hmox1 | heme oxygenase (decycling) 1 | 1.3 | 6.7e-05 |
| Dnajb1 | DnaJ (Hsp40) homolog, subfamily B, member 1 | 1.5 | 8.1e-08 |
| Retsat | retinol saturase (all trans retinol 13,14 reductase) | 0.92 | 4.7e-05 |
| Fkbp4 | FK506 binding protein 4 | 0.64 | 5.2e-05 |
| Dnaja1 | DnaJ (Hsp40) homolog, subfamily A, member 1 | 1.2 | 6.8e-05 |
| Spsb1 | splA/ryanodine receptor domain and SOCS box containing 1 | 1.4 | 3.8e-06 |
| Slc3a1 | solute carrier family 3, member 1 | -1.23 | 1.7e-07 |
| Acot1 | acyl-CoA thioesterase 1 | 2.95 | 5.6e-05 |
| Ddit3 | DNA-damage inducible transcript 3 | 0.85 | 9.6e-06 |
| Chordc1 | cysteine and histidine-rich domain (CHORD)-containing 1 | 1.23 | 1.5e-05 |
| Acaa1b | acetyl-Coenzyme A acyltransferase 1B | 2.32 | 7.4e-05 |
| Hsp90ab1 | heat shock protein 90 alpha (cytosolic), class B member 1 | 0.84 | 3.7e-06 |
| Oit3 | oncoprotein induced transcript 3 | 0.68 | 4.2e-05 |

D. In SHR treated with salsalate versus SHR treated with placebo

None
